# Male mice treated with combined anti-fibrotic therapeutics, IPW5371 and tadalafil, are predisposed to adverse cardiovascular events

**DOI:** 10.3389/fphar.2025.1537494

**Published:** 2025-04-02

**Authors:** Jazz Q. Stephens, Uriel Blas-Machado, Chrissy Sherrill, David Caudell, Nancy Kock, Ashley M. Davis, Jordyn M. Whitfield, Barry Hart, Kylie Kavanagh

**Affiliations:** ^1^ Department of Pathology, Wake Forest School of Medicine, Winston-Salem, NC, United States; ^2^ Department of Pathology, College of Veterinary Medicine, University of Georgia, Athens, GA, United States; ^3^ Department of Population Health and Pathobiology, North Carolina College of Veterinary Medicine, Raleigh, NC, United States; ^4^ Division of Comparative Medicine and Department of Pathology and Laboratory Medicine, University of North Carolina, Chapel Hill, NC, United States; ^5^ StageBio, Mt. Jackson, VA, United States; ^6^ Innovation Pathways, Palo Alto, CA, United States; ^7^ College of Health and Medicine, University of Tasmania, Hobart, TAS, Australia

**Keywords:** aortopathy, vasculopathy, anti-fibrosis agents, irradiation, high-fat and high-fructose diet, drug interaction, sex differences

## Abstract

Fibrosis is a pathological process with few therapeutic options. Experimental molecules are being developed to counteract the fibrotic effects through TGFβ receptor inhibition. Additionally, phosphodiesterase 5 (PDE5) inhibitors also have anti-fibrotic effects; however, the mechanism of action remains unresolved. IPW5371 is an example of an experimental TGFβ-mediated anti-fibrotic compound, and tadalafil is an example of a PDE5 inhibitor. Irradiation increases the frequency of fibrotic lesions, driven by the activation of the TGFβ pathway. We hypothesized that the TGFβ receptor and PDE5 inhibitor agents would be additive in their ability to prevent fibrosis development in tissues in a sub-lethal whole-body irradiation mouse model. However, the combined use of anti-fibrotic agents, tadalafil and IPW5371, caused increased male mouse mortality associated with ascending and thoracic aortic rupture compared to mice that only received one of the drugs. Following histopathological analysis of the mouse hearts, we also observed that irradiation protected against lesions caused by the combination therapy as non-irradiated male mice had significantly worse outcomes as compared to irradiated male mice, substantiating the drug–drug interaction independent of the radiation effects. This important drug interaction needs further investigation as these agents are developed for anti-fibrosis therapy, and PDE5 inhibitors are commonly prescribed to male patients.

## Introduction

Diseases characterized by excessive and progressive fibrosis involve the abnormal accumulation of collagen-rich connective tissue. This process disrupts normal tissue architecture and, if allowed to persist, will impair the ability of the affected organ to operate effectively. In conditions such as pulmonary fibrosis, liver cirrhosis, delayed effects of radiation exposure, and systemic sclerosis, excessive fibrosis plays a central role in disease progression and can be life-threatening if not managed (Rosenbloom et al., 2017; Schultz-Hector and Trott, 2007). There are ongoing efforts in biomedical research to find effective therapeutics to treat diseases that result from fibrosis as options for patients are currently extremely limited. The primary canonical pathway known to drive genes that initiate collagen formation and deposition is transforming growth factor beta (TGFβ) receptor signaling. Rational drug development to target fibrosis-induced organ dysfunction, thus, could target TGFβ signaling and angiogenesis pathways; however, the pleiotropic roles that TGFβ-signaling plays in cell functions, including neoplastic processes and normal immune cell processes, have raised concerns.

New molecules targeting TGFβ-signaling are in active development, for example, the small-molecule inhibitor of TGFβ receptor 1 kinase, IPW5371, which has been demonstrated, in previous studies, to mitigate the delayed pro-fibrotic effects of radiation. Important consequences of irradiation (de Vathaire et al., 2012; Holmqvist et al., 2014; Meacham et al., 2009; van Nimwegen et al., 2014; Bacarella et al., 2019; Kavanagh et al., 2015; Thakur et al., 2021) are modulated via canonical TGFβ receptor signaling, which is a key pathway for fibrosis and has an active role in radiation damage to multiple organs (Schultz-Hector and Trott, 2007; Fanning et al., 2017; Rabender et al., 2016; DeBo et al., 2016). It has been demonstrated that cardiopulmonary, hepatic, and renal functions can be protected by IPW5371 following radiation exposure in rodents (Rabender et al., 2016; Fish et al., 2023; Szalanczy et al., 2024). Furthermore, the small-molecule inhibitor of the TGFβ-receptor 1, activin-like receptor kinase 5 (ALK5), has shown efficacy in maintaining vascular endothelial function in the blood–brain barrier (Senatorov et al., 2019).

Alternative pharmacological strategies for combating fibrosis are also under investigation. For example, phosphodiesterase 5 (PDE5) inhibitors, noted for their widespread clinical use for erectile dysfunction, can restore capillary growth in multiple tissues in multiple *in vivo* animal models (Lee et al., 2015; Nio et al., 2017) and have an anti-fibrotic effect that is independent of canonical TGFβ signaling (Kang et al., 2011).

We hypothesized that progressive fibrosis could be inhibited by compounds in these aforementioned classes, and the effects would be additive or synergistic. Herein, we model excess fibrosis by employing ionizing radiation, which can release TGFβ1 from its latent complex to its active form, which then binds to a complex of type I and type II receptors (TGFβR1 and 2) on many cell types, triggering a cascade that leads to the expression of pro-fibrotic genes and proteins. We have demonstrated that tissue levels of TGFβ were still higher in nonhuman primates (NHPs) years post-exposure (Fanning et al., 2017); however, the application of this NHP model is limited due to available animals, their costs, and limits on access. Rodent models are preferred for discovery and assessment of investigational drugs; they show tissue fibrosis and capillary rarefication, as do NHPs (Seemann et al., 2014; Jin et al., 2014). In a rodent preclinical model, we report cardiovascular injury associated with administration of TGFβR1 kinase inhibitors administered in *combination* with a PDE5 inhibitor in irradiated and un-irradiated male mice fed a Western-style diet. This report aims to document these adverse outcomes in a drug combination that has previously not been reported.

## Material and methods

### Animal studies

All mouse experimental procedures were performed according to the guidelines of state and federal laws, the U.S. Department of Health and Human Services, and the Wake Forest University School of Medicine (WFUSM) Institutional Animal Care and Use Committee. Mice ([IACUC; Protocol #A15-068] C57BL/6N, Envigo, Frederick, MD) were kept in a light-controlled room (12-h light and dark cycles). All procedures were described and reviewed by the WFUSM IACUC in protocol #A18-024. Husbandry was enhanced with added nesting material and low placement on the housing rack to reduce exposure to light and minimize stress. Mice were fed a Western diet (protein = 17.3% of calories, lipids = 34.9% of calories, and carbohydrates = 47.7% of calories) (Table 1). Diet conditions mimic current intakes, and the addition of saturated fats and simple carbohydrates drives relevant inflammatory processes and enhances the translational relevance of study conditions. Diet intake was estimated on a day each month by calculating the difference between the amount of diet fed and that which remained after 24 h/cage and averaged per total gram weight of mouse/cage. Water was available *ad libitum*. Mice were acclimated to the diet for 4 weeks prior to irradiation and acidified, antibiotic-supplemented water (pH 2.5–3, enrofloxacin 25 mg/mL) for 1 week prior to and then an additional 2 weeks following irradiation/sham. Euthanasia at predetermined study endpoints was achieved by inducing a deep plane of anesthesia using isoflurane inhalation (SomnoSuite, Kent Scientific), followed by thoracotomy.

**TABLE 1:** Dietary nutrient breakdown for the Western diet-fed to mice in studies 1, 2, and 3.

Dietary component	
Cholesterol (mg/calorie)	0.38
Protein (% of calories)	17.34
Lipid (% of calories)	34.93
Carbo (% of calories)	47.74
Fructose (% of calories)	11.00
Simple CHO (% of calories)	22.08
FATTY ACIDS	% of fat
Saturated (%)	38.32
Monounsaturated (%)	34.38
Polyunsaturated (%)	17.00
Sodium (mg/kcalorie)	0.30

**FIGURE 1:**
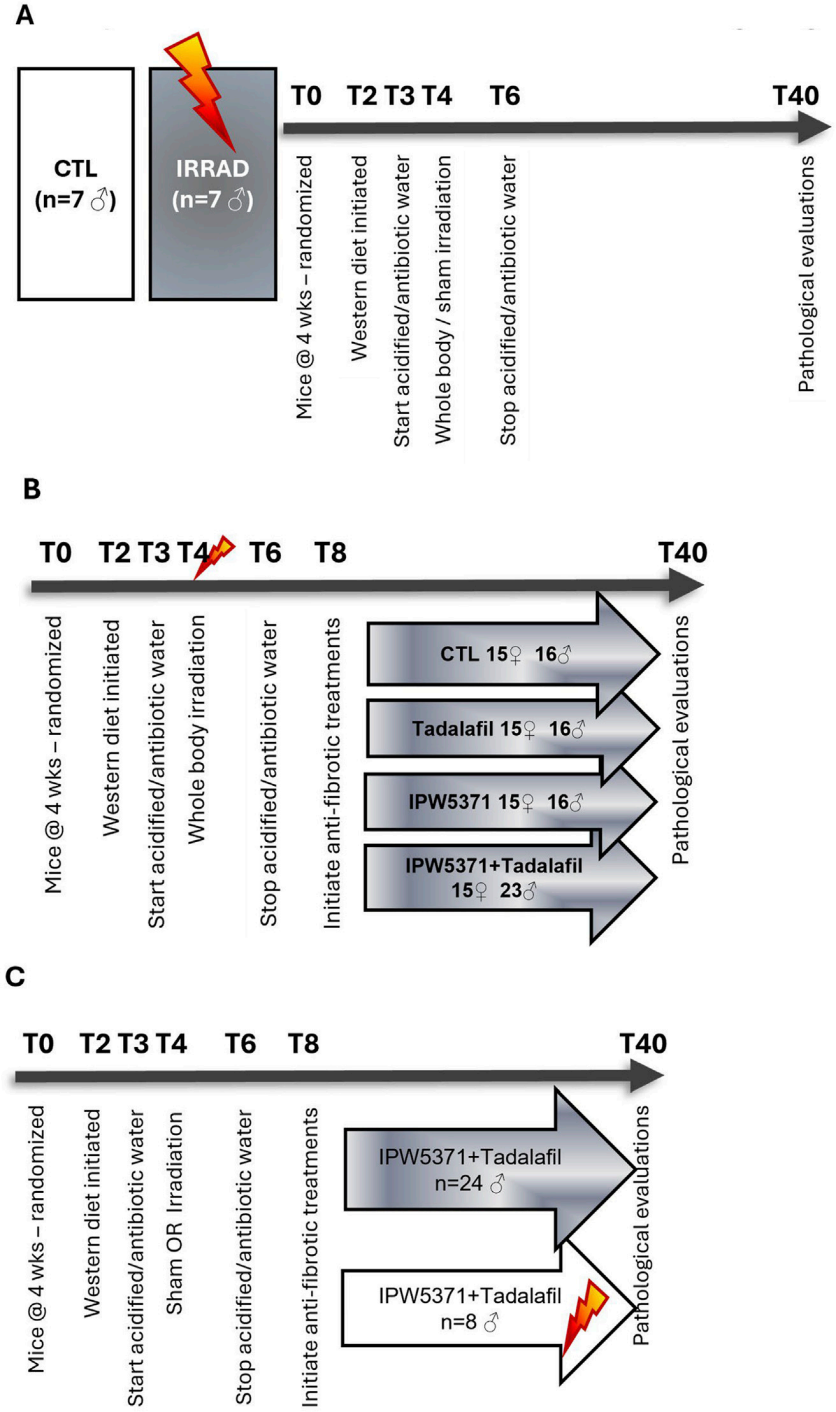
Overview of study designs. Timing (T) of the study is indicated by the numerical marking of weeks (0–40). **(A)** Study 1: confirmation of irradiation-induced pro-fibrotic signaling. Mice were exposed to Western diet and sham or sub-lethal whole-body irradiation (7Gy) to induce persistent inflammation and fibrosis. **(B)** Anti-fibrotic therapeutic intervention study. Mice were randomized into treatment groups to evaluate anti-fibrotic agents (tadalafil and/or IPW5371 or control) to inhibit diet- and irradiation-induced effects. **(C)** Anti-fibrotic intervention evaluation in non-irradiated male mice. Follow-up study to assess the effects of the drug interaction in non-irradiated male mice.

Study 1: confirmation of irradiation-induced pro-fibrotic signaling (Figure 1A). Male mice (n = 8/group, C57BL/6N, Envigo, Frederick, MD) were exposed to our total-body irradiation (TBI) protocol, or sham, which was sub-lethal and had evidence of delayed effects of radiation present at 6 months post-exposure (Szalanczy et al., 2024). We delivered a 7 Gray (Gy) total dose as two 3.5 Gy fractions delivered at 0.358 Gy/min with less than 10 min between the fractions. Radiation was emitted from a Co^60^ source (3 × 6,500 Ci; J.L Shepherd & Assoc., Model 484R2, S.N. 7,210). Mice were anesthetized with a combination of xylazine and ketamine and turned from ventral to dorsal recumbency between fractions to ensure even body exposure. Low-dose irradiation (IRRAD) was administered slowly to the entire body based on observations that irradiation reliably induces cellular senescence and sub-lethal irradiation accelerates the incidence of chronic diseases (Kavanagh et al., 2015; DeBo et al., 2016; Azzam et al., 2012; Ito, 1994; Lopez-Otin et al., 2013). The irradiation protocol resulted in 0% lethality, and <5% of mice showed acute clinical signs that called for a single day of fluid or analgesic support. Evaluations were conducted at 6 months post-irradiation.

Study 2: anti-fibrotic therapeutic intervention study (Figure 1B). Male and female C57BL/6N mice (n = 131, equal sex representation) were included in our intervention study. At 12 weeks of age, mice were randomly assigned to one of four treatment groups: irradiated mice treated either with a novel TGFßR1 kinase inhibitor (IPW5371, Innovation Pathways Inc., n = 15 female and n = 16 male mice) at 30 mg/kg/d, a phosphodiesterase 5 inhibitor (tadalafil [TAD], 5 mg/kg/d, n = 15 female and n = 16 male mice), both in combination (IPW5371+TAD; n = 15 female and n = 23 male mice), or control conditions (CTL, n = 15 female and n = 16 male mice). Control mice were irradiated and diet-exposed but had no exposure to anti-fibrotic compounds. Irradiation was as described for study 1. Two weeks following TBI and recovery, mice began receiving a modified Western diet, which had tadalafil and IPW5371 compounded in it to achieve the daily doses based on 5g/25 g mouse food intake. The supplied diet/administered treatment was increased across the duration of study as the mice gained weight. All pathological evaluations were conducted after 6 months post-irradiation as a period where symptoms of the acute radiation syndrome are resolved and delayed effects present as predominant morbidities.

### Anti-fibrotic interventions

**FIGURE 2:**
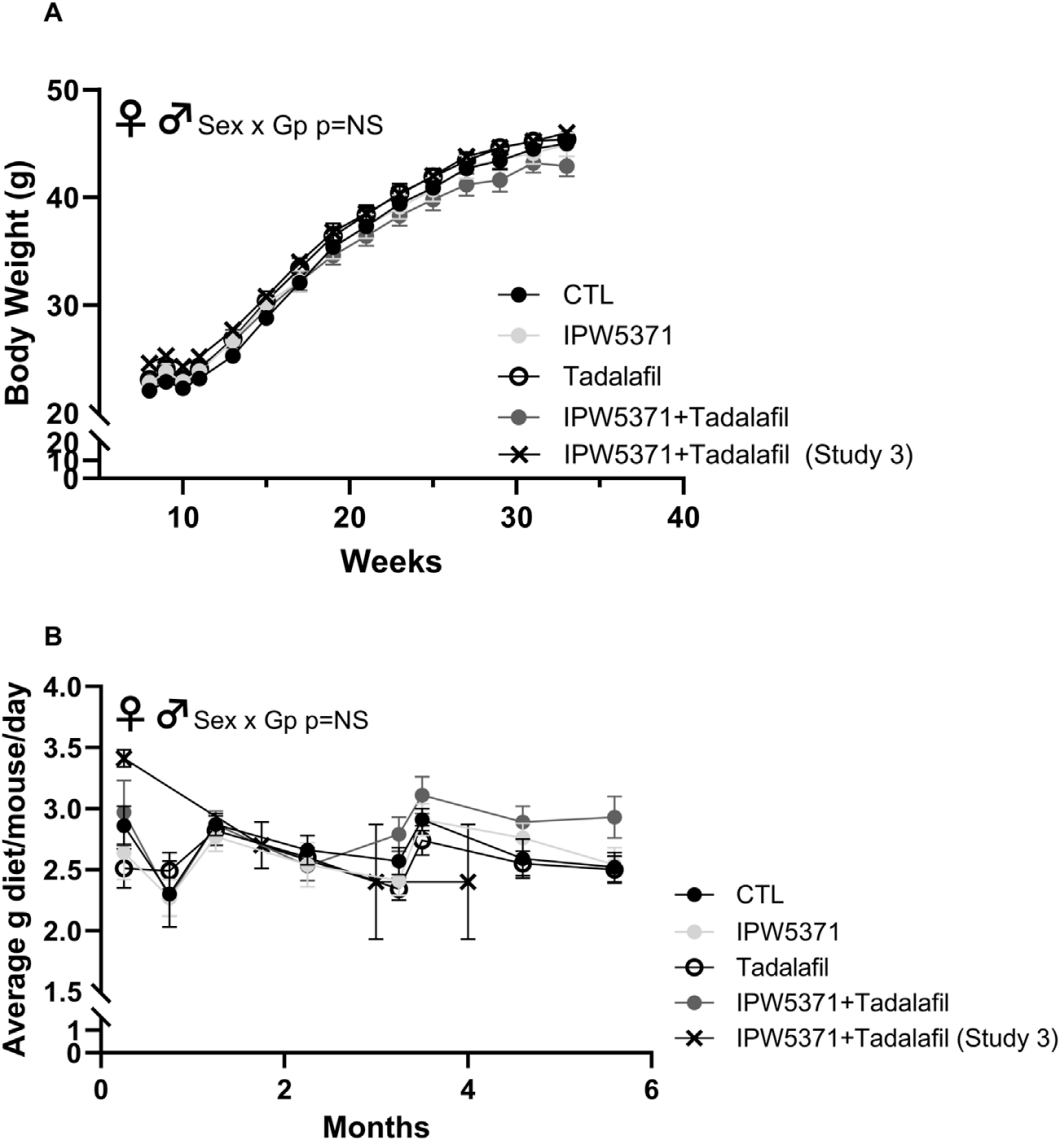
**(A)** Body weight in grams and **(B)** food intake as average grams/mouse/day was comparable across all groups of mice in studies 1 and 3, which is indicative that drug exposure did not change caloric consumption and weight gain. There were no significant sex by group interactions (NS = non-significant at p > 0.05), and so male and female mice are shown as combined data in these graphs. Means with standard error bars are shown. GP, Group.

The TGFβR1 kinase inhibitor, IPW5371, is in the developmental stage as a small-molecule radiation medical countermeasure (Fish et al., 2023). IPW5371 is an inhibitor of the TGFβ pathways. Currently, TGFβ antagonists, including the small-molecule inhibitor of TGFβRI kinase, galunisertib, are in clinical trials for a variety of cancers. We used approximately 30 mg/kg/d, assuming equal consumption of diet among the group-housed mice. The dose was selected based on increased survival and decreased lung and liver injury observed in irradiated mice (Rabender et al., 2016; Szalanczy et al., 2024). Tadalafil is an FDA-approved pharmaceutical agent for erectile dysfunction and benign prostatic hypertrophy. It is a phosphodiesterase 5 inhibitor (PDE5), which has positive effects on tissue perfusion. PDE5 inhibition restores exercise-activated perfusion in aged muscle through improved nitric oxide signaling (Nyberg et al., 2015a; Nyberg et al., 2015b), improves perfusion in clinical cases of muscular dystrophy (Martin et al., 2012), and may also reduce extracellular matrix deposition, as seen in renal disease models (Lee et al., 2015). We deployed 5 mg/kg/d tadalafil based on prior reports, and it is expected to approximate 20 mg/day administered to people (Saad et al., 2016). Figure 2 documents the body weights and average food consumption of mice in studies 2 and 3 to substantiate that drug exposure through the dietary formulation did not alter food intake or the resultant body weight gains over time. No differences in food consumption or weight gains between male and female mice were detected.

Study 3: anti-fibrotic intervention in non-irradiated male mice (Figure 1C): A follow-up study was performed to confirm that the adverse effects seen were independent from the irradiation (IRRAD) status. Non-irradiated male mice (n = 8) and irradiated male mice (n = 24 mice) were kept in identical housing, diet, and care conditions, including the treated water and sham irradiation procedure, and were all exposed to IPW5371 and tadalafil for 6 months.

### Histopathological analysis

Only mice euthanized at the pre-determined study end date (6 months post TBI and/or anti-fibrotic drug exposure) were evaluated. The heart and its base, lung, and liver were collected and fixed in 4% paraformaldehyde for 24 h prior to trimming, routine tissue processing, and paraffin embedding. Samples were also collected from animals found dead prior to the scheduled study end date. Sections of approximately 5 μm thickness were made and stained with hematoxylin and eosin for histopathological evaluations and Masson’s trichrome (MTC) stain for collagen. Blinded to the data, board-certified veterinary pathologists (UBM and NK) evaluated the slide sections and reported the results prior to interpretation of data relative to experimental grouping. The ascending aorta and heart were examined for the presence or absence of significant tissue alterations (lesions) such as, but not limited to, inflammation, fibrosis, necrosis, and hemorrhage (Berridge et al., 2016). Lesion incidence, severity, and distribution were recorded. If present, histopathologic scores were assigned as grades 0 (histologically normal), 1 (minimal = a focal, subtle, or trivial change), 2 (mild = an easily identifiable change of limited severity and/or distribution), 3 (moderate = an obvious change with normal tissue remaining), or 4 (severe = an extensive/maximal change that obliterates much of the normal tissue) based on an increasing extent and/or complexity of change, unless otherwise specified. Lesion distribution was recorded as focal, multifocal, or diffuse, with distribution scores of 1, 2, or 3, respectively. All histopathological research studies were conducted in accordance with the US FDA Good Laboratory Practice regulations (21 CFR Part 58 and subsequent amendments). Tissues stained positively with MTC were quantitated by digital pathological image analysis and expressed as the percentage of the total tissue area (Visiopharm Corporation, Westminster, CO).

### Tissue measures

Total TGFβ levels were measured in study-1 mice. We collected the liver after 6 months of irradiation or sham irradiation to confirm that delayed pathological processes were still active at this chronic stage. Values were generated by enzyme-linked immunosorbent assay (R&D Systems, DY1697-05) and normalized to liver total protein, as measured by bicinchoninic acid assay (Thermo Fisher Scientific, 23227). The liver was chosen as it is central and deep in the rodent, and the outcomes were considered indicative of fibrosis activation. Activation of the TGFβ pathway was measured by immunoblotting lung tissue for phosphorylation of SMAD2/3 (Abcam, #ab63399) and shown as expression relative to glyceraldehyde 3-phosphate dehydrogenase (GAPDH; Abcam #ab9485). We measured the plasma and tissue (lung) concentrations of IPW5371 at both 10 and 30 mg/kg at trough concentrations (24 h post-dose) as previously described (Rabender et al., 2016).

### Data analysis

Data were log-transformed if required to achieve statistical assumptions of normality. Data are expressed throughout as means ± standard error of the mean, unless otherwise indicated. Group differences were analyzed using analysis of variance (ANOVA), with the group and sex included as model variables. If sex was not a significant variable, the assessment was conducted as one-way ANOVA. The alpha level was set at ≤0.05 for statistical significance. Trends are defined as alpha set at ≤0.10. Post hoc analyses were conducted using Tukey’s Honest Significant Differences testing. Correlation coefficients were determined by Pearson’s r statistics for association. Differences in proportions were assessed by chi-squared statistics. All statistical testing was performed using Statistica V10 (StatSoft Inc., Carlsbad, CA).

## Results

**FIGURE 3:**
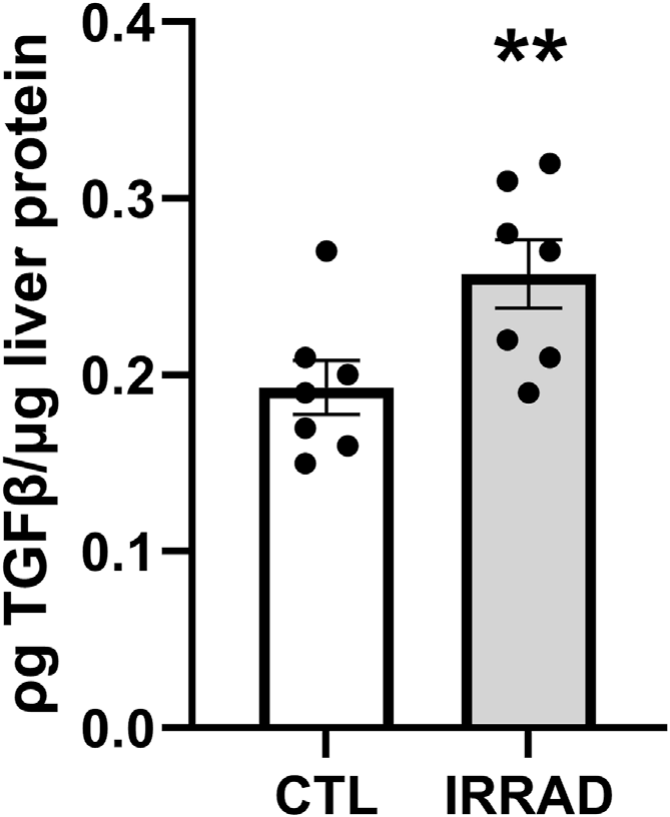
Sub-lethal whole-body irradiation (7Gy) leads to persistently increased transforming growth factor β (TGF-β) in the liver 6 months after exposure. Means with standard error bars are shown. N = 7/group; **p < 0.01.

Study 1: sub-lethal irradiation led to persistent upregulation of fibrosis signaling with 24% higher TGFβ levels in the liver 6 months after exposure (Figure 3, p < 0.01). At this time, all mice were obese and showed no clinical signs of disease (data not shown).

**FIGURE 4:**
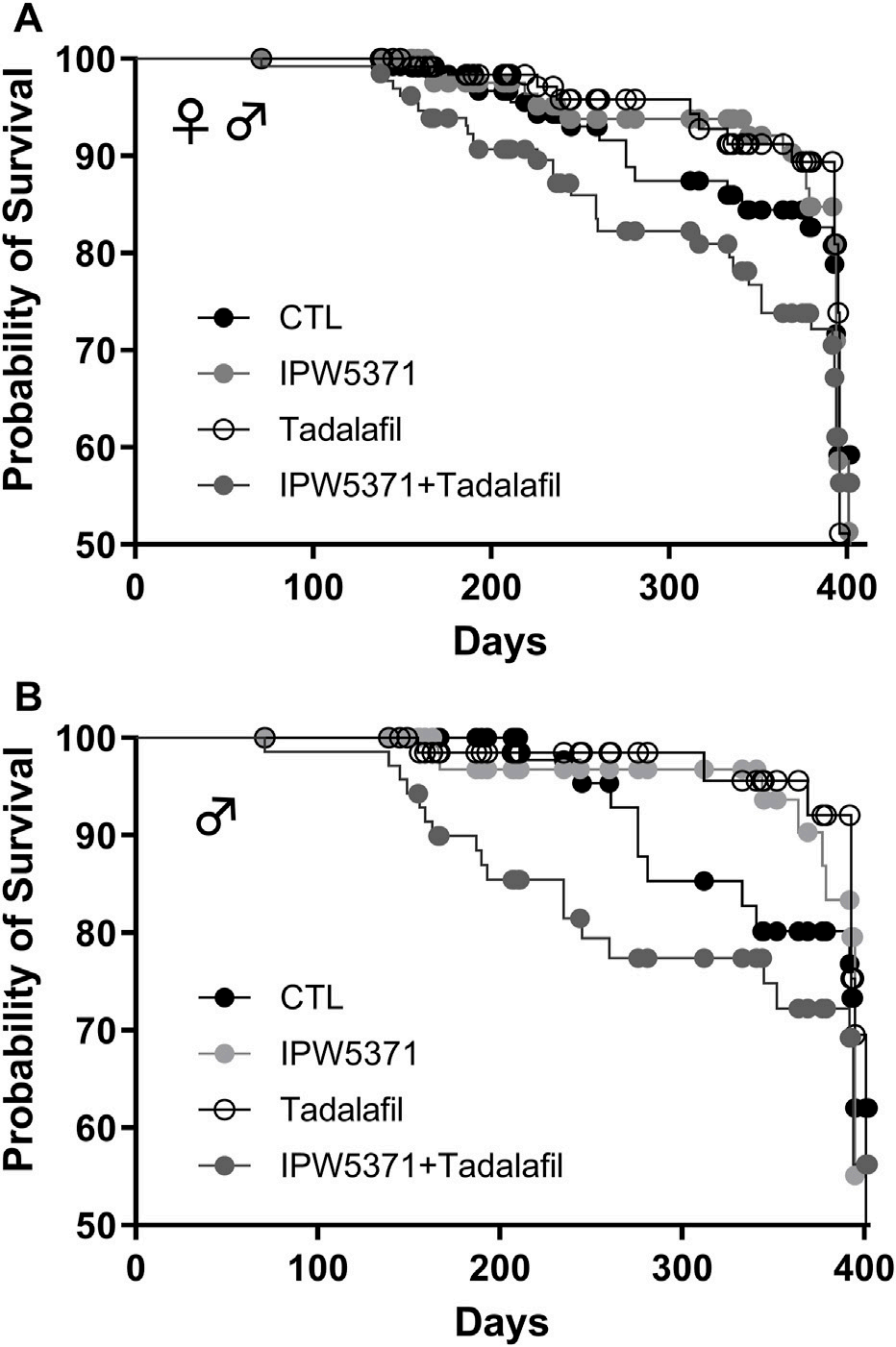
Survival curves for irradiated mice kept on Western diet and treated with experimental anti-fibrotic therapies. Sub-lethal whole-body irradiation was performed on day 30 of diet consumption, and the study end was 365 days later. Pathological outcomes were measured in a subset evaluated at 180 days. **(A)** Male and female mice together showed that the combination of IPW5371 and tadalafil caused a significantly higher mortality (p < 0.001). **(B)** The excess risk of death was sex-specific, with only male mice exposed to this drug combination having a significantly higher probability of death (p < 0.001). Female mice had no group differences in survival (data not shown).

**FIGURE 5:**
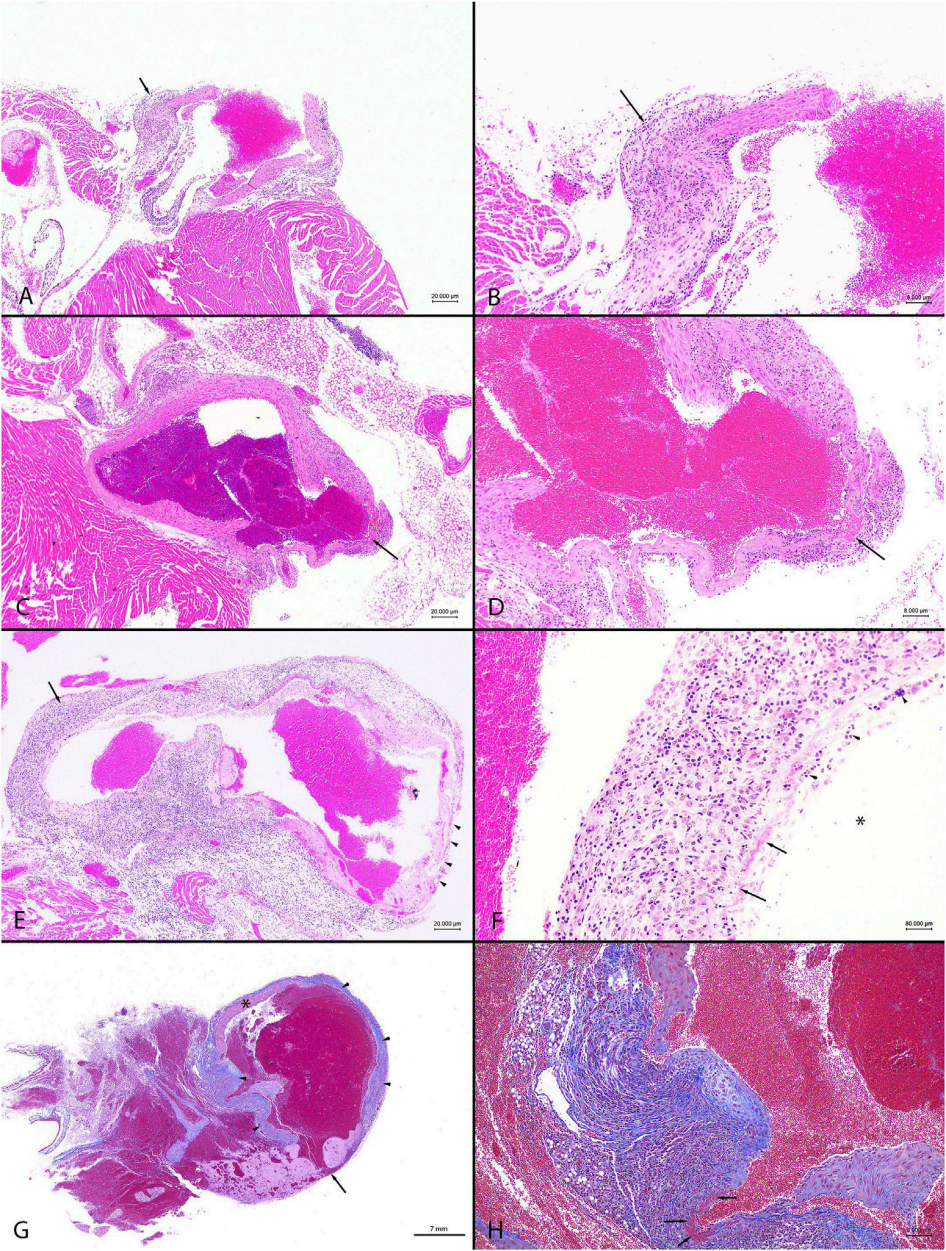
Representative images from observed cardiovascular pathology in male C57BL/6N mice. The combined administration of anti-fibrotic therapeutics IPW5371 (TGFβ-mediated anti-fibrotic drug) and tadalafil (PDE5 inhibitor) led to increased mortality associated with ascending and thoracic aortic inflammation, fibrosis, and rupture compared to mice that only received one of the drug treatments. Abbreviations: HE = hematoxylin and eosin. MTC = Masson’s trichrome **(A)**. Animal #105, tadalafil + IPW5371 treatment. Heart base, ascending aorta, and inflammation. The focally thickened and hypercellular wall (arrow) has transmural fibroplasia with a mixed inflammatory cellular infiltrate. HE stain. Scale bar = 20 μm. **(B)** Animal #105, tadalafil + IPW5371 treatment. Heart base, ascending aorta, inflammation. Higher magnification of **(A)**. widely spaced, mixed inflammatory cellular infiltrate within a loosely arranged fibroblastic tissue stroma shows obscured thickened vessel wall (arrow). HE stain. Scale bar = 8 μm. **(C)** Animal #168, tadalafil + IPW5371 treatment. Heart base, aorta, inflammation with necrosis. The arrow shows a focus of necrosis and thinning of the vessel wall. HE stain. Scale bar = 20 μm. **(D)** Animal #168, tadalafil + IPW5371 treatment. Heart base, aorta, inflammation. Higher magnification of **(C)** a layer of hypereosinophilic material (fibrin; arrow) mixed with necrotic cellular debris and mixed inflammatory cellular infiltrate shows smudging of the thin vessel wall. HE stain. Scale bar = 8 μm. **(E)** Animal #167, tadalafil + IPW5371 treatment. Heart base, aorta, and inflammation. Transmural mixed inflammatory cellular infiltrates obscure the walls of the vessel (arrow). A thin layer of fibrin covers the thin-walled vessel (arrowheads). HE stain. Scale bar = 20 μm. **(F)** Animal #167, tadalafil + IPW5371 treatment. Heart base, aorta, and inflammation. Higher magnification of the wall of the aorta in **(E)** loosely arranged, mixed inflammatory cells and spaced fibroblasts shows obscured vessel wall. A thin layer of fibrin (arrows) mixed with necrotic cellular debris (arrowheads) covers the denuded endothelial surfaces (arrow). The asterisk (*) is in the lumen of the vessel. HE stain. Scale bar = 8 μm. **(G)** Animal #110, tadalafil + IPW5371 treatment. Thoracic aorta, rupture. There is focal rupture (arrow) of the aorta. From the borders of the ruptured vessel and along the adjacent, irregularly thickened walls of the aorta, there is widespread fibroplasia (arrowheads, blue color). The asterisk (*) is on the wall of the unaffected part of the vessel. MTC stain. Scale bar = 7 mm. **(H)** Animal #110, tadalafil + IPW5371 treatment. Thoracic aorta, rupture. Higher magnification of the wall of the aorta in **(G)** widely spaced, mixed inflammatory cellular infiltrate within a loosely arranged fibroblastic tissue stroma (blue color) shows obscured disrupted vessel walls. The arrows point to a focus of fibrinous necrosis. MTC stain. Scale bar = 8 μm.

Study 2: the health observations in study 1 were consistent with observations in study 2 at 6 months, where there were no group differences in the body weight among control or drug-treated mice (Table 2). Target organ weights are shown, and no effect of IPW5371 was reported. Tadalafil-treated mice had lungs that weighed less at necropsy. Survival curves indicate that mice exposed to the combination of IPW5371 and tadalafil had higher mortality than mice in all other treatment groups, including groups of mice exposed to each agent as single exposures (Figure 4A; p < 0.001 for median survival time) and that this was driven predominantly by mortality in male mice (Figure 4B; p < 0.001 for median survival time). Mice exposed to IPW5371 or tadalafil as single agents had improved survival as compared to control mice, indicative of the specific nature of a drug interaction on survival. Male mice treated with both tadalafil and IPW5371 were consistently found dead with gross evidence of severe intrathoracic hemorrhage or hemothorax, which correlated on histopathology to thinning and rupture of the ascending aorta (Figure 5). No female mice died prior to 6 months from any hemorrhagic process. These unexpected deaths were observed exclusively in male mice exposed to both anti-fibrotic agents initiated 57 days after initiation of drug treatment, and a similar gross pathology was observed throughout all phases of the study. Decedent mice were photographed, but tissues were not collected or used in any analyses. Irradiated mice that were not exposed to any anti-fibrotic agents were euthanized for humane reasons, and prior to the scheduled 6-month evaluations, they predominantly had gross evidence of neoplasia (lymphoma affecting liver, spleen, or thyroid). Frequencies of neoplasia by gross examination across all groups in study 2 were 6% in CTL, 26% in tadalafil, 16% in IPW5371%, and 26% in IPW5371 + tadalafil.

**TABLE 2:** Bodyweight (BW), organ weight, and tissue fibrosis in sub-lethally whole-body irradiated mice kept on a Western diet and treated with anti-fibrotic therapy (study 2; n = 6–8/group). Mean values are shown with the standard error value in parentheses. No sex differences in any outcomes were observed. No interactive effects of drug agents were observed in accumulation of Type 1 collagen at 6 months, as measured by Masson’s trichrome (MTC) positive staining area. A reduction in liver collagen was observed in mice exposed to TGF-β receptor 1 inhibition with IPW5371.

Bodyweight (g)	CTL	IPW3571	Tadalafil	IPW3571+ tadalafil
Male (n = 14–16/group)	46.0 (0.42)	47.2 (1.48)	46.0 (0.90)	44.1 (1.56)
Female (n = 14–16/group)^§^	43.8 (1.31)	42.5 (1.47)	44.8 (0.86)	41.6 (0.97)
Heart fibrosis (% area MTC^+ve^)	1.87 (0.50)	2.24 (1.21)	1.30 (0.40)	1.51 (0.35)
Lung fibrosis (% area MTC^+ve^)	7.63 (2.88)	8.65 (3.53)	7.63 (2.88)	6.95 (3.11)
Liver fibrosis (% area MTC^+ve^)	0.18 (0.07)	0.10 (0.04)*	0.18 (0.07)	0.13 (0.06)*
Heart weight (% BW)	0.32 (0.01)	0.40 (0.03)	0.34 (0.03)	0.38 (0.04)
Lung weight (%BW)	0.41 (0.03)	0.45 (0.03)	0.32 (0.01)^**#**^	0.46 (0.04)
Liver weight (%BW)	5.68 (0.41)	4.94 (0.27)	5.94 (0.72)	5.02 (0.24)

*p < 0.05 for the effect of IPW3571; ^**#**^p < 0.005 for the effect of tadalafil compared to all other groups. ^**§**^Effect of sex on BW, p = 0.03.

On histopathologic analysis, rupture of the ascending aorta correlated with gross pathology observation of the hemothorax. The ruptured vessels had evidence of thinning of the vessel walls covered and/or replaced by a layer of fibrin. The adjacent, hypercellular vessel walls were obscured by scattered, mixed inflammatory cellular infiltrates within a fibroblastic stroma, with foci of fibrinous necrosis. Findings or observations were detected in the ascending aorta and coronary vessels, with extension of inflammatory infiltrates into the adjacent myocardium. Representative images of the lesion types observed in the study mice are shown in Figure 5. All other findings were considered incidental. The numbers of animals, samples, and frequency of lesions reported from study 2 are tabulated (Table 3). Lesions described as inflammatory or necrotic were exclusively observed in mice exposed to TGFβR1 inhibition. Lesion severity grading from study 2 mice tissues is shown in Table 4.

**TABLE 3:** Summary of histopathological findings (lesion frequencies) in the cardiovascular tissues of sub-lethally study 2 irradiated mice that were exposed to anti-fibrotic agents. Inflammation and necrosis were exclusively observed in IPW5371-exposed mice. Lesion grading is shown in Table 4.

Organ	Lesion	Group Name	Number of animals (both sexes	Number of samples examined	Number of animals with lesions
**Ascending aorta and pulmonary artery**	Inflammation	CTL	9	9	0
		IPW5371	8	7	0
		Tadalafil	10	9	0
		IPW5371 + Tadalafil	14	12	3
	Lymphocytic infiltrates	CTL	9	9	1
		IPW5371	8	7	1
		Tadalafil	10	9	1
		IPW5371 + Tadalafil	14	12	1
	Degeneration	CTL	9	9	0
		IPW5371	8	7	0
		Tadalafil	10	9	0
		IPW5371 + Tadalafil	14	12	0
	Necrosis	CTL	9	9	0
		IPW5371	8	7	1
		Tadalafil	10	9	0
		IPW5371 + Tadalafil	14	12	0
	Fibroplasia	CTL	9	9	0
		IPW5371	8	7	0
		Tadalafil	10	9	1
		IPW5371 + Tadalafil	14	12	3
**Heart and coronary arteries**	Inflammation	CTL	9	9	0
		IPW5371	8	8	1
		Tadalafil	10	10	0
		IPW5371 + Tadalafil	14	14	1
	Lymphocytic infiltrates	CTL	9	9	3
		IPW5371	8	8	0
		Tadalafil	10	10	0
		IPW5371 + Tadalafil	14	14	0
	Degeneration	CTL	9	9	9
		IPW5371	8	8	8
		Tadalafil	10	10	10
		IPW5371 + Tadalafil	14	14	14
	Necrosis	CTL	9	9	0
		IPW5371	8	8	2
		Tadalafil	10	10	0
		IPW5371 + Tadalafil	14	14	1
	Fibroplasia	CTL	9	9	1
		IPW5371	8	8	1
		Tadalafil	10	10	0
		IPW5371 + Tadalafil	14	14	0

**TABLE 4:** Lesion type and severity grade in cardiovascular tissues of irradiated mice from study 2, which were treated with anti-fibrotic agents for 6 months.

Organ	Lesion	Group Name	Number of animals with lesions	Number of animals per degree of lesion severity*			
				Minimal	Mild	Moderate	Severe
**Ascending aorta and pulmonary artery**	Inflammation	CTL	0	0	0	0	0
		IPW5371	0	0	0	0	0
		Tadalafil	0	0	0	0	0
		IPW5371 + Tadalafil	3	0	1	1	1
	Lymphocytic infiltrates	CTL	1	1	0	0	0
		IPW5371	1	1	0	0	0
		Tadalafil	1	1	0	0	0
		IPW5371 + Tadalafil	1	1	0	0	0
	Degeneration	CTL	0	0	0	0	0
		IPW5371	0	0	0	0	0
		Tadalafil	0	0	0	0	0
		IPW5371 + Tadalafil	0	0	0	0	0
	Necrosis	CTL	0	0	0	0	0
		IPW5371	1	0	0	0	1
		Tadalafil	0	0	0	0	0
		IPW5371 + Tadalafil	0	0	0	0	0
	Fibroplasia	CTL	0	0	0	0	0
		IPW5371	0	0	0	0	0
		Tadalafil	1	0	1	0	0
		IPW5371 + Tadalafil	3	0	2	1	0
**Heart and coronary arteries**	Inflammation	CTL	0	0	0	0	0
		IPW5371	1	0	0	0	1
		Tadalafil	0	0	0	0	0
		IPW5371 + Tadalafil	1	0	1	0	0
	Lymphocytic infiltrates	CTL	3	3	0	0	0
		IPW5371	0	0	0	0	0
		Tadalafil	0	0	0	0	0
		IPW5371 + Tadalafil	0	0	0	0	0
	Degeneration	CTL	9	9	0	0	0
		IPW5371	8	8	0	0	0
		Tadalafil	10	10	0	0	0
		IPW5371 + Tadalafil	14	12	0	2	0
	Necrosis	CTL	0	0	0	0	0
		IPW5371	2	0	2	0	0
		Tadalafil	0	0	0	0	0
		IPW5371 + Tadalafil	1	0	1	0	0
	Fibroplasia	CTL	1	0	0	1	0
		IPW5371	1	0	0	0	1
		Tadalafil	0	0	0	0	0
		IPW5371 + Tadalafil	0	0	0	0	0

* Lesion severity grade: 0 (histologically normal); 1 (minimal = a focal, subtle, or trivial change); 2 (mild = an easily identifiable change of limited severity and/or distribution); 3 (moderate = an obvious change with normal tissue remaining); or 4 (severe = an extensive/maximal change that obliterates much of the normal tissue) based on an increasing extent and/or complexity of change, unless otherwise specified.

**FIGURE 6:**
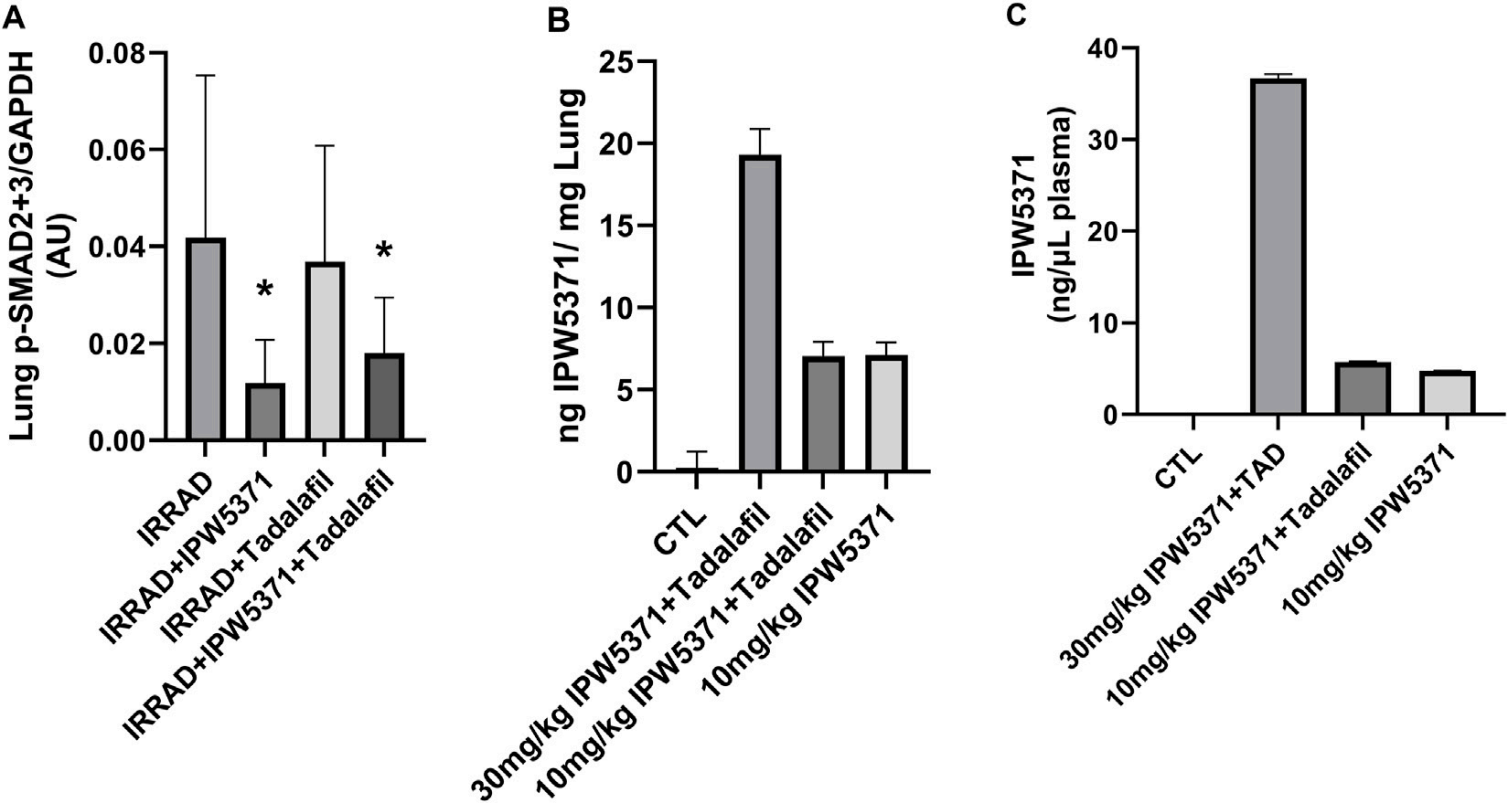
No pharmacokinetic (PK) or pharmacodynamics interaction was observed between anti-fibrotic compounds. **(A)** TGF-β pathway inhibition, measured by activation of SMAD2/3, was selectively reduced with IPW5371 (30 mg/kg; male and female mice n = 6–8/group). **(B)** Tissue concentrations of IPS5371 were dose-proportional at 10 and 30 mg/kg doses with no effect of tadalafil co-administration (male mice, n = 4–6/group). **(C)** Plasma concentrations of IPW5371 confirm no PK interaction of agents (male mice, n = 4–6/group). Means with standard error bars are shown.

We evaluated the pharmacokinetics and pharmacodynamics of our anti-fibrotic compounds (Figure 6) considering the liver and lung canonical TGFβ levels. We additionally measured levels of the novel compound IPW5371 in plasma. We observed a significant reduction in phosphorylated SMAD2/3 in lung tissue with TGFβR1 inhibition using IPW5371 (p < 0.05 for the effect of IPW5371), with values approximating half of that of mice that were irradiated and/or exposed to PDE5 inhibition. No sex effect was present with respect to pathway inhibition. There was no interactive effect of PDE5 inhibition and no further reduction in TGFβ pathway activation via this pathway. Tissue concentrations of IPW5371 showed a dose-proportional increase in the lung steady-state drug levels, with 30 mg/kg IPW5371 being almost exactly three times higher than 10 mg/kg IPW5371. Furthermore, co-administration of agents did not change the tissue distribution (Figures 6A, B). Plasma concentrations of IPW5371 were measured; 30 mg/kg achieved therapeutic concentrations based on prior efficacy in irradiated rodents (Rabender et al., 2016), and no evidence of a pharmacokinetic interaction was observed, with IPW5371 concentrations being equivalent in the presence and absence of tadalafil (Figure 6C).

**FIGURE 7:**
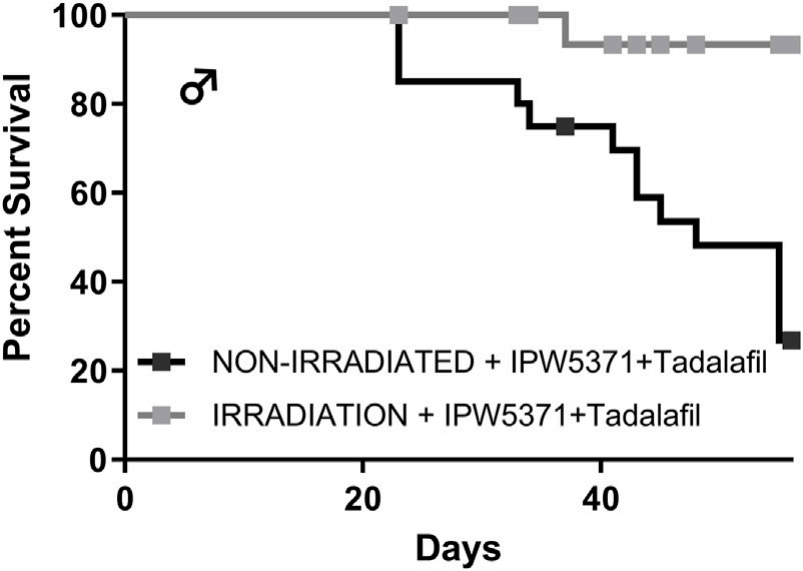
The combination of anti-fibrotic agents was more lethal in control Western diet-fed male mice (p < 0.001) as compared to similarly treated mice that had sub-lethal whole-body irradiation.

Study 3: the adverse effects on the cardiovascular integrity that resulted from co-administration of PDE5 and TGFβR1 inhibition in irradiated mice were confirmed to be generalized and not specific to the irradiated mouse model. Non-irradiated male mice compared to irradiated male mice had significantly worse survival while undergoing the same experimental protocol (Figure 7; p < 0.001 for median survival). Un-irradiated mice dosed only with IPW5371 or tadalafil were not evaluated. Mortality was observed in less than 14 days after drug exposure was initiated, and histological lesions were replicated to those seen in irradiated mice (Table 5). Frequencies of lesions in drug-treated mice were significantly higher for inflammation and mixed cell infiltrates in the great vessels, heart, and coronary arteries. The frequency of lesion severity is tabulated in Table 6. We speculate that the pro-fibrotic processes initiated by irradiation may have delayed and reduced the detrimental effects of the anti-fibrotic drug combination, leading to better survival rates in the first 100 days post-radiation exposure (Figures 4B, 6).

**TABLE 5:** Frequency of lesions in the cardiovascular tissues of study 3 non-irradiated control mice that were exposed to anti-fibrotic agents. These mice had worsened survivorship. Significantly higher frequencies of inflammation and lymphocytic infiltrates were seen in IPW5371-exposed mice.

Organ	Lesion	Group Name	Number of animals	Number of samples examined	Number of animals with lesions	P-value
**Ascending aorta and pulmonary artery**	Inflammation	CTL	8	6	1	
		IPW5371 + Tadalafil	21	15	8	<0.001
	Lymphocytic infiltrates	CTL	8	6	0	
		IPW5371 + Tadalafil	21	15	1	<0.001
	Degeneration	CTL	8	6	0	
		IPW5371 + Tadalafil	21	15	0	-
	Necrosis	CTL	8	6	0	
		IPW5371 + Tadalafil	21	15	0	-
	Fibroplasia	CTL	8	6	0	
		IPW5371 + Tadalafil	21	15	4	<0.001
**Heart and coronary arteries**	Inflammation	CTL	8	8	1	
		IPW5371 + Tadalafil	21	21	6	0.005
	Lymphocytic infiltrates	CTL	8	8	0	
		IPW5371 + Tadalafil	21	21	2	0.002
	Degeneration	CTL	8	8	8	
		IPW5371 + Tadalafil	21	21	21	-
	Necrosis	CTL	8	8	0	
		IPW5371 + Tadalafil	21	21	0	-
	Fibroplasia	CTL	8	8	0	
		IPW5371 + Tadalafil	21	21	0	-

**TABLE 6:** Lesion type and severity grade in cardiovascular tissues of study 3 non-irradiated mice, which were treated with anti-fibrotic agents for 6 months.

Organ	Lesion	Group Name	Number of animals	Number of samples examined	Number of animals per degree of lesion severity*			
					Minimal	Mild	Moderate	Severe
**Ascending aorta and pulmonary artery**	Inflammation	CTL	8	6	0	0	0	0
		IPW5371 + Tadalafil	21	15	0	6	2	0
	Lymphocytic infiltrates	CTL	8	6	0	0	0	0
		IPW5371 + Tadalafil	21	15	0	0	0	0
	Degeneration	CTL	8	6	0	0	0	0
		IPW5371 + Tadalafil	21	15	0	0	0	0
	Necrosis	CTL	8	6	0	0	0	0
		IPW5371 + Tadalafil	21	15	0	0	0	0
	Fibroplasia	CTL	8	6	0	0	0	0
		IPW5371 + Tadalafil	21	15	0	4	0	0
**Heart and coronary arteries**	Inflammation	CTL	8	8	0	0	0	0
		IPW5371 + Tadalafil	21	21	0	3	2	0
	Lymphocytic infiltrates	CTL	8	8	0	0	0	0
		IPW5371 + Tadalafil	21	21	0	0	0	0
	Degeneration	CTL	8	8	0	0	0	0
		IPW5371 + Tadalafil	21	21	0	4	0	0
	Necrosis	CTL	8	8	0	0	0	0
		IPW5371 + Tadalafil	21	21	0	0	0	0
	Fibroplasia	CTL	8	8	0	0	0	0
		IPW5371 + Tadalafil	21	21	0	0	0	0

* Lesion severity grade: 0 (histologically normal); 1 (minimal = a focal, subtle, or trivial change); 2 (mild = an easily identifiable change of limited severity and/or distribution); 3 (moderate = an obvious change with normal tissue remaining); or 4 (severe = an extensive/maximal change that obliterates much of the normal tissue) based on an increasing extent and/or complexity of change, unless otherwise specified.

## Discussion

We aim to report an unanticipated drug–drug interaction in male mice, leading to significant mortality. Notably, single-agent exposure to the ALK5 inhibitor IPW5371 and the PDE5 inhibitor tadalafil had no adverse effects on survival and may have a small protective effect (p > 0.05). Similar lesions described herein have been previously observed in rodents dosed with ALK5 inhibitors such as galunisertib; however, modifications of dosing strategies with galunisertib have afforded an exposure paradigm that is safer for humans (Fujiwara et al., 2015; Santini et al., 2019).

ALK5 phosphorylates SMAD proteins after binding of TGFβ to its receptor (TGFβR1), initiating nuclear translocation and pro-fibrotic gene transcription. ALK5 inhibitors have been developed for use in several disease processes including oncology, hepatic cirrhosis, and pulmonary hypertension. Our study replicates prior reports of ALK5 inhibition in demonstrating a reduction in SMAD activation and fibrosis signaling; however, it also replicates findings of cardiovascular lesions (Anderton et al., 2011; Dave et al., 2018a). We extend prior reports by documenting a male sex-specific effect of this pathway and a significant pathological interaction with PDE5 inhibition, which is a commonly prescribed drug to male patients. Furthermore, we confirm that the interaction effects on the cardiovascular tissues are not limited to C57Bl/6N mouse models with radiation exposure (Unthank et al., 2015).

The implications and importance for human health need further investigation by way of dose-ranging studies. We confirm that PDE5 inhibitor treatment did not alter the absorption, distribution, or metabolism of the experimental agent that reduces TGFβR1 activity, nor did it alter the downstream pharmacodynamic effects on SMAD2/3. PDE5 inhibitors are considered to have potential as anti-fibrotic agents (Nio et al., 2017; Tantini et al., 2005; Gong et al., 2014) and have the additional benefit of promoting neovascularization (Martin et al., 2012). Our study did not observe tadalafil-related reductions in lung pSMAD levels in the lung tissue. However, it is notable that at the time of this writing, there were three case reports of arterial dissection in human patients following exposure to single doses of a PDE5 inhibitor; all cases were characterized by being male patients, application of a range of drug use profiles (approximating 0.1–4 mg/kg, single and chronic dosing), and acute hemorrhage, following aortic, cerebral, or vertebral artery rupture, leading to death (Anadol Kelleci et al., 2017; Dersch et al., 2013; Lameijer et al., 2012). We believe that the effects of PDE5 inhibition are additive to TGFβR1 inhibition, as we observed deaths in our mice treated with IPW5371 alone, albeit at lower frequencies. Predicted drug–drug interactions using compound structures and *in silico* databases (Ivanov et al., 2018) between galunisertib, as a drug class representative, and tadalafil indicates no overall significant increased risk of drug–drug interactions across classes of severity. However, potential effects on hepatic CYP1A2 leading to minor pharmacodynamic effects by moderate changes in hepatic metabolism and potential reduction in renal excretion are predicted. It is, therefore, possible that altered tadalafil concentrations resulted and future studies should include circulatory concentrations of both pharmacological agents. This predictive result from two currently prescribed agents underscores the importance of the reported cardiovascular lesions in this study.

The lesions described replicate closely those reported with ALK5 inhibitors in rodents, characterized by inflammation and necrosis, seen in heart valves and large arteries (Berridge et al., 2016; Anderton et al., 2011). However, cardiovascular lesions after exposure to similar IPW5371 and radiation doses were not observed in a prior study (Rabender et al., 2016). Study-related differences include the following: 1) the strain of mice, 2) field and dose of radiation, and 3) human-relevant diet. The current study diet is lower in fat than most Western diets designed for rodents; however, it includes important proinflammatory components of cholesterol, fructose, and saturated fatty acids at human-relevant levels (Clapper et al., 2013). The study mice exposed to putative fibrosis inhibitors in this dietary context had rapid lesion development, leading to more significant mortality than in mice that were additionally exposed to radiation. Our model shows that non-irradiated mice had lower TGFβ in liver tissue; therefore, if this is generalized, then irradiation-induced vascular fibrosis may lead to protection from drug-induced loss of integrity. There are even reports in hypercholesterolemic mice that low-dose irradiation can have an anti-inflammatory effect in heart tissue (Mathias et al., 2015), which may be in effect.

Mechanisms that modify vascular inflammation are described with ALK5 and TGFβ inhibition, and data support both positive and negative effects (Liu et al., 2022; Zhou et al., 2022). The embryonic knock-out model of ALK5 deficiency demonstrates vascular dilation with fewer pericytes and collagen fibers in developing arteries (Dave et al., 2018b) in mature male mice with knock-out of TGFβR1. Female mice were not examined to confirm a sex-specific effect, but the similarities are striking. The mechanism for aortic aneurysm in TGFβR1 knock-out mice was suggested to be dysregulation of cyclophilin A (CypA), as inhibition of this enzyme was able to eliminate lethal lesions and reduce aortic dilation (Zhou et al., 2019). The target cell types implicated in this, and other studies, are vascular smooth muscle cells (Zhou et al., 2022). Studies targeting reduction in TGFβ signaling in vascular smooth muscle cells only were adequate to recapitulate the lesions we see, indicating that this cell type is critical in maintaining vessel wall integrity (Hu et al., 2015). Considering that blockage of TGFβ is a potential therapeutic strategy for aortopathies in people (Rosenbloom et al., 2017), the results presented here are pertinent.

Inflammatory mechanisms for PDE5 inhibition are less clear as anti-inflammatory effects have been reported with therapeutic use (Nio et al., 2017). More importantly, additive reduction in structural collagen deposition in these high-pressure cardiovascular locations at the heart base is likely (Tantini et al., 2005; Dave et al., 2018b) as this class of drugs has been reviewed in rats for effects further down the pressure gradient at the level of the aorta (Teixeira et al., 2006). All agents in the PDE5 class induce reliable vasodilation, whereby the key target cell type is vascular smooth muscle cells. Vasorelaxation with PDE5 occurs even when the nitric oxide and cyclic AMP pathways are inhibited, suggesting that additional inhibitory mechanisms are active. These off-target activities on relaxation are undefined, but the classic inhibition of nitric oxide and cyclic AMP pathways did reverse the anti-proliferative effects observed with PDE5 inhibition (Tantini et al., 2005). Together, these PDE5 and TGFβ receptor inhibited activities may lead to a vascular wall with fewer smooth muscle cells that are able to produce the needed collagens for integrity, leading to degeneration and inflammation.

Sex differences in inflammation are well-appreciated with the suppressive effects of estrogen demonstrated in many preclinical models and clinical diseases. Specific to the vasculature of cardiovascular disease rodent models, female mice have shown lower artery inflammation and greater fibrosis of plaques (Monslow et al., 2020). Furthermore, in rodent models of aneurysm and inflammation, male mice were more prone to lesions and increased lesion severity (Porritt et al., 2020). In our study, no female mouse was found dead, and while lesions in female mice were histologically present, the frequency and severity were less than those detected in male mice.

This study describes the importance of the vascular lesions observed and mortality seen with co-administration of drug agents in development and in current use. However, the study has notable limitations. Some endpoints did have small sample sizes, which are disclosed in the legends, and due to limited blood volumes, we could not measure sex hormones. Female mice were smaller than male mice, so we believe that irradiation did not eliminate ovarian function, but estrogen would be an additional data point to confirm that differences were related to sex steroids (Porritt et al., 2020; Marek et al., 2017). Future studies should include sex hormones and check if survival curves differ in castrated male mice to further substantiate the sex-dependent mechanisms behind mortality. The difference in mortality between irradiated and non-irradiated male mice also supports the need to look at radiation effects on sex hormones in male mice as known sex differences in radiosensitivity and TGFβ have been documented (Irons et al., 2012). Future studies could include the evaluation of lesions that develop in standardized low-fat, low-carbohydrate, cholesterol-free laboratory chow diets; however, these diets are often enriched in protective isoflavones (Singh and Seed, 2020) and lack the proinflammatory components of Western diets. The vascular pathology we observed was consistent with what had been reported previously for ALK5 inhibitors, but the combination of tadalafil and IPW5371 producing a greater number of cardiovascular events was unexpected. The collection of the heart and arteries was not optimized for pathological evaluations, including measures of smooth muscle cell distributions across the treatment groups. Finally, lower doses of inhibitors and effects on TGFβ levels in irradiated and non-irradiated mice would confirm pharmacodynamics actions and whether safe levels exist, and, as mentioned already, concurrent measurements of IPW5371 and tadalafil alone and in combination are needed to understand drug–drug interactions. The case reports relating to singular PDE5-related aneurysms are, however, cautionary. Modification of the dose schedule and dosing levels of tadalafil and IPW5371 could optimize the efficacy and minimize side effects. The Human Protein Atlas indicates that TGFβR1 is more highly expressed in the liver and lungs than in connective tissues, whereas rodent distributions are unknown. The distributions in humans indicate that this cautionary report has relevance.

In summary, we present a new diet plus low-dose irradiation mouse model to study anti-fibrosis therapies. The mice have an ongoing evidence of TGFβ pathway activation, as measured by pSmad2/3, and this pathway is functionally inhibited by IPW5371, an ALK5 inhibitor. In this model, we established that IPW5371 and tadalafil each have neutral or improved effects on survival, but when administered together, the combination decreases survival compared to untreated animals. Moreover, we identified an important drug interaction with PDE5 inhibitor, tadalafil, which needs to be further studied to understand the clinical relevance.

## Data Availability

The raw data supporting the conclusions of this article will be made available by the authors, without undue reservation.
